# Glycolysis-associated lncRNAs in cancer energy metabolism and immune microenvironment: a magic key

**DOI:** 10.3389/fimmu.2024.1456636

**Published:** 2024-09-13

**Authors:** Xi Zhang, Yunchao Zhang, Qiong Liu, Anqi Zeng, Linjiang Song

**Affiliations:** ^1^ School of Medical and Life Sciences, Chengdu University of Traditional Chinese Medicine, Chengdu, Sichuan, China; ^2^ Translational Chinese Medicine Key Laboratory of Sichuan Province, Sichuan Academy of Chinese Medicine Sciences, Sichuan Institute for Translational Chinese Medicine, Chengdu, Sichuan, China

**Keywords:** cancer, glycolysis, immune, lncRNA, metabolism

## Abstract

The dependence of tumor cells on glycolysis provides essential energy and raw materials for their survival and growth. Recent research findings have indicated that long chain non-coding RNAs (LncRNAs) have a key regulatory function in the tumor glycolytic pathway and offer new opportunities for cancer therapy. LncRNAs are analogous to a regulatory key during glycolysis. In this paper, we review the mechanisms of LncRNA in the tumor glycolytic pathway and their potential therapeutic strategies, including current alterations in cancer-related energy metabolism with lncRNA mediating the expression of key enzymes, lactate production and transport, and the mechanism of interaction with transcription factors, miRNAs, and other molecules. Studies targeting LncRNA-regulated tumor glycolytic pathways also offer the possibility of developing new therapeutic strategies. By regulating LncRNA expression, the metabolic pathways of tumor cells can be interfered with to inhibit tumor growth and metastasis, thus affecting the immune and drug resistance mechanisms of tumor cells. In addition, lncRNAs have the capacity to function as molecular markers and target therapies, thereby contributing novel strategies and approaches to the field of personalized cancer therapy and prognosis evaluation. In conclusion, LncRNA, as key molecules regulating the tumor glycolysis pathway, reveals a new mechanism of abnormal metabolism in cancer cells. Future research will more thoroughly investigate the specific mechanisms of LncRNA glycolysis regulation and develop corresponding therapeutic strategies, thereby fostering new optimism for the realization of precision medicine.

## Introduction

Cancer is the most prevalent form of malignant tumor, resulting from genetic mutations that induce uncontrolled cell growth and division (1–3). Metabolic reprogramming is the distinctive biochemical signature shown by cancer cells, one of the “cancer hallmarks” (4, 5), which allows cancer cells to adapt to the microenvironment through plasticity and high flexibility in nutrient acquisition and utilization, particularly metabolic changes associated with key steps in cancer progression, such as proliferation, the initiation of metastasis, circulation, and colonization (6). This metabolic phenotype is typified by a preference for using glycolysis as a source of non-aerobic energy (7). This peculiar metabolic occurrence is frequently called the “Warburg effect” (8, 9). LncRNAs actively regulate the cancer epigenome and are significant participants in the regulation of the Warburg effect (10). The observation that a significant proportion of lncRNAs are specifically expressed in tumors and can regulate a  diverse array of tumor biological processes has led to lncRNAs showing great promise in tumor therapy, especially lncRNA-mediated glucose metabolism signaling pathways and transcription factors, highlighting important recent insights into the mechanisms regulating cancer metabolism (11–13). Notwithstanding these recent advancements, there exists an insufficient comprehension of cancer cell metabolism, with a particular dearth of research in the domain of LncRNA-mediated regulation of cancer-associated metabolic reprogramming pertaining to glycolysis.

Long non-coding RNAs (lncRNAs) comprise a class of RNA molecules distinguished by their length in excess of 200 nucleotides and lack the ability to encode proteins. They may participate in a vast array of physiopathological processes associated with tumors. Their mechanisms of action include: i) lncRNAs control cellular processes by competitively adsorbing, as well as down-regulating miRNAs via base complementation. ii) lncRNAs attach to epigenetically associated proteins and modify their post-transcriptional translation. iii)lncRNAs can influence target gene expression through chromatin modifiers and transcription factors, among other regulatory proteins. iv) lncRNAs are capable of interacting with and modulating the activity of proteases. V) lncRNAs are capable of regulating cellular processes. VI) Triplets of lncRNAs can influence the transcription of certain genes when they bind to genomic DNA (14–17).

**Figure 1:**
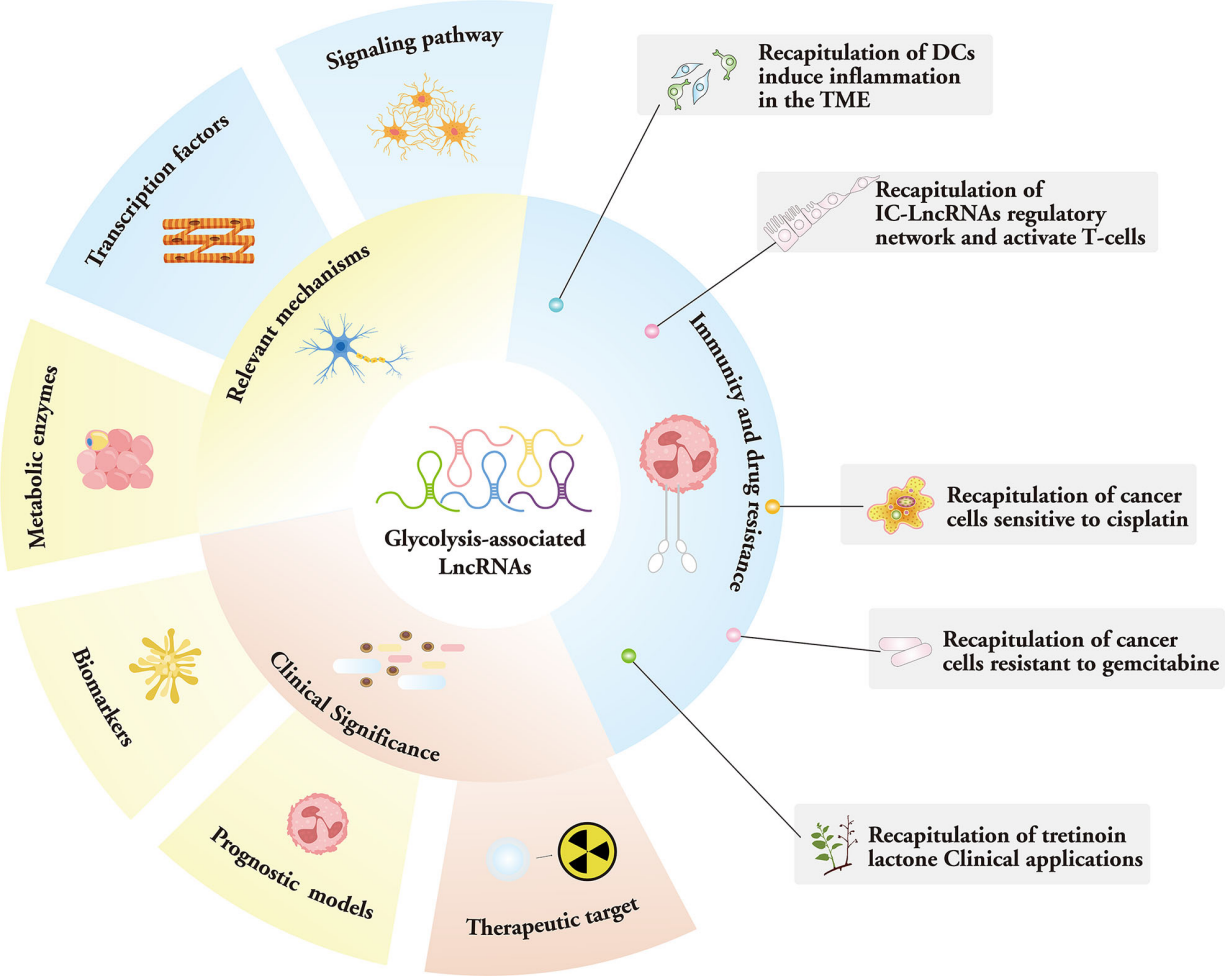
Mechanism, function, and clinical significance of glycolysis-associated lncRNAs.

Recently, a genome-wide analysis of the human cancer transcriptome revealed that lncRNA expression is among the most prevalent transcriptional changes observed in cancer (18, 19). Consolidating data also suggest that chemoresistance is largely determined by the amount of ATP inside cells, and that lncRNAs can function as efficacious therapeutic targets for an array of malignancies (20). In this paper, we review the mechanisms of LncRNA in the tumor glycolytic pathway and their potential therapeutic strategies, including current alterations in cancer-related energy metabolism with lncRNA mediating the expression of key enzymes, lactate production and transport, and the mechanism of interaction with transcription factors, miRNAs, and other molecules. By regulating LncRNA expression, the metabolic pathways of tumor cells can be interfered with to inhibit tumor growth and metastasis, thus affecting the immune and drug resistance mechanisms of tumor cells. Furthermore, we discuss the clinical importance of these lncRNAs, including potential application scenarios as therapeutic biomarkers (21) (**Figure 1**).

## Mechanisms governing the function of glycolysis-associated lncRNAs in cancer cells

Glucose metabolism, which includes processes such as oxidative phosphorylation, glucose uptake, and lactate production, serves as a crucial energy supply for cancer cells. Cancer cells alter glucose metabolism to encourage the development, survival, and medication resistance of tumor cells. LncRNAs exhibit aberrant expression across a broad spectrum of cancer cell types and are essential for a number of cancer’s hallmark characteristics (22, 23). LncRNAs play regulatory roles by means of interaction with proteins, mRNAs, ncRNAs, and DNA, serving as numerous functional molecules in numerous cellular processes, linked to cancer, such as signals, decoys, scaffolds, and guides (24). Here, we summarize how lncRNAs regulate glucose metabolism by modulating relevant signaling pathways, as well as mediating the activities of metabolism-associated transcription factors and enzymes, with an emphasis on understanding the mechanisms that govern the metabolism of malignancy.

### Glycolysis-associated lncRNAs regulate cancer energy metabolism involved in signaling pathways

Alterations in metabolism-related proteins are correlated with modifications in metabolic pathways, which ultimately become carcinogenic signaling pathways or are triggered by oncogenes lncRNAs that can indirectly regulate metabolic pathways through post-translational modifications. Among the results noted, functional modifications in the adenosine monophosphate-activated protein kinase (AMPK) pathway, the metastasis-associated lung adenocarcinoma (LA) transcript 1 (MALAT1) pathway, and the Hippo/YAP pathway are particularly noticeable.

#### AMPK

As a significant energy sensor, AMPK is indispensable for regulating biological energy metabolism. AMPK activation can enhance glucose metabolism and antioxidant synthesis to control metabolic reprogramming and redox balance in response to metabolic stress. Recent research indicates that numerous signaling mechanisms regulate AMPK phosphorylation epigenetically, including LA-associated MACC1-AS1, mesenchymal stem cell-induced lncRNA HCP5, and UNC5B antisense lncRNA 1 (UNC5B-AS1). A widely known oncogene, MACC1, is the transcriptional regulator from which MACC1-AS1 is an antisense lncRNA transcript. Another study found that MACC1-AS1 overexpression promoted hexokinase 2 (HK2) and lactate dehydrogenase A (LDHA) activities, decreased reactive oxygen species (ROS) levels, and a nicotinamide adenine dinucleotide/nicotinamide adenine dinucleotide phosphate (NADP+/NAPDH) ratio, the upregulation of MACC1-AS1 was observed to enhance the expression of MACC1 in glioma cells through the activation of the AMPK pathway, hence exerting influence on glucose metabolism and redox balance, exerting a tumor-promoting effect (25). Furthermore, MSC-induced lncRNA HCP5 stimulates fatty acid oxidation through the miR-3619-5p/AMPK/PGC1α/CEBPB axis, promoting stemness and chemoresistance in gastric cancer (26). Suppressing UNC5B-AS1 can reduce the development and spread of colon cancer by upregulating the expression of miR-622 and blocking the AMPK and PI3K/AKT pathways (27). AMPK is a crucial regulator of glucose metabolism in tumor cells; it can reduce the energy supply of tumor cells by inhibiting the glycolytic pathway and transporter protein expression, while decreasing antioxidant capacity, thereby diminishing metabolic plasticity.

#### MALAT1

The lncRNA known as MALAT1 has been well recognized as a pivotal contributor to the progression of cancer. The lncRNA MALAT1 also engages in competitive interactions using natural microRNAs to adjust the downstream gene expression levels (28). Knockdown of MALAT1 inhibited MYBL2 expression and reduced the level of mammalian target of rapamycin (mTOR) pathway phosphorylation. The inhibition of MALAT1 and MYBL2 or the administration of rapamycin, a mTOR pathway inhibitor, resulted in a substantial suppression of prostate cancer (PCa) cell growth and a notable decrease in the Warburg effect. In addition, up-frameshift protein 1(UPF1) may regulate MALAT1, and the UPF1/MALAT1 pathways might potentially serve as a promising area of therapeutic exploration for the treatment of gastric cancer. The levels of UPF1 expression in healthy lung tissues were higher than in human LA tissues, suggesting that a reduction in nonsense-mediated decay (NMD) contributes to LA formation. UPF1 has additionally been documented as a tumor suppressor (29, 30). Another study found that, in combination with the translation process of lncRNA MALAT1 as well as its manifestation in cancer cells, the association between lncRNA-MALAT1 polymorphisms and carcinogenesis should be revealed. Nevertheless, it is imperative to validate this idea at subsequent stages by specific experiments. As an illustration, by studying the lncRNA-MALAT1’s inhibitory effect on metabolites and assessing the outcomes by measuring the spread or proliferation of cancer cells, the study of MALAT1 is important in inhibiting the proliferation of tumor cells.

#### Hippo/YAP

The Hippo/YAP pathway plays a pivotal role in various metabolic processes within the context of cancer, encompassing cell viability, proliferation, angiogenesis, and energy metabolism. Some lncRNAs, such as LINC00857, GHET1, and TNRC6C-AS1, are closely connected to the regulation of energy metabolism and yes-associated protein (YAP) phosphorylation in cancer. It has been shown that LncRNA GHET1 promotes hypoxia-induced triple-negative breast cancer (TNBC) proliferation, invasion, and glycolysis via the Hippo/YAP signaling pathway (31). Mechanistically, YAP was retained in the cytoplasm when lncRNA GHET1 was inhibited, leading to a rise in LATS1 and YAP phosphorylation levels. Conversely, hypoxia or overexpression of the lncRNA GHET1 promoted the growth of TNBC and the nuclear translocation of YAP. However, Lin et al. (32) demonstrated that ovarian cancer glycolysis and progression were controlled by LINC00857 by Spiking miR-486-5p, which up-regulated YAP1 and hence regulated the Hippo signaling pathway, which may potentially offer novel insights for therapeutic approaches targeting ovarian cancer. More information and mechanisms by which glycolysis-associated lncRNAs regulate cancer through signaling pathways are shown in **Table 1**.

**Table 1:** Glycolysis-associated lncRNAs regulate cancer through signaling pathways.

LncRNA	Level	Cancer type	Mechanisms	Glycolysis	Biological function	Ref.
MACC1- AS1	↑	Glioma	AMPK	Activates	Proliferation, apoptosis, metabolic plasticity	(25)
HCP5	↑	Gastric cancer	miR-3619-5p/AMPK/PGC1α/CEBPB	Activates	Promote stemness, chemo-resistance	(26)
UNC5B-AS1	↑	Colorectal cancer	miR-622/AMPK/PI3K/AKT	Activates	Tumor growth, metastasis	(27)
MALAT1	↓	Prostate Cancer	MALAT1/MYBL2/mTOR	Suppresses	intracellular acidification, apoptosis	(29)
MALAT1	↑	NSCLC	miR-613	Activates	Proliferation, colony formation, apoptosis	(115)
GHET1	↑	Triple-Negative Breast Cancer	Hippo/YAP	Activates	Proliferation, invasion	(31)
LINC00857	↑	Ovarian cancer	Hippo	Activates	Proliferation, migration, invasion, suppresses apoptosis	(32)

The arrows denote upregulation or downregulation in cancer.

In conclusion, Hippo-YAP signaling both regulates and is regulated by metabolism, thereby making a significant contribution to cellular metabolism. Consequently, the Hippo/YAP signaling network is utilized in a variety of environments and can influence the development of pancreatic β-cells involved in glucose metabolism, lipid metabolism, and autophagy. There are numerous putative LncRNA-mediated pathways associated with glycolysis, such as H19, NF-KB, and PTEN, the mechanisms of which remain unknown. We have only provided summaries of three representative signaling pathways. The correlation between intricate and adaptable signaling pathways and the energy metabolism of tumors must be substantiated through additional experiments.

### Glycolysis-associated lncRNAs regulate cancer energy metabolism of transcription factors.

Considering that lncRNAs influence the regulation of targets by transcription factors, they also regulate the reprogramming of energy metabolism via glycolysis-associated transcription factors. LncRNAs exert regulatory control over the activity of these proteins via processes such as hydroxylation, phosphorylation, and ubiquitination, which enhances the manifestation of enzymes associated with metabolic processes. Additionally, lncRNAs facilitate the malignant evolution of neoplastic growths. Henceforth, we shall proceed to deliberate upon the recently documented mechanism of regulating representative factors (33).

#### HIF-1α

In rapidly growing tumor tissues, hypoxia-inducible factor-1α (HIF-1α) performs an essential function in the metabolic reprogramming of tumor cells through the stimulation of gene transcription that encodes glycolytic enzymes and glucose transporters. For example, HIF-1α stimulates the transcription of lncRNA RAET1K via miR-100-5p to control the glycolysis induced by hypoxia in hepatocellular carcinoma cells (34). Furthermore, by creating a positive feedback loop involving AKT/HIF-1α signaling, the overexpression of lncRNA CASC9 under hypoxic settings enhances glycolysis and the epithelial-mesenchymal transition in pancreatic cancer (35, 36).

Interestingly, tumor-associated macrophages (Tam) deliver bone marrow-specific lncRNA through extracellular vesicles. HIF-1α-stabilizing long-stranded lncRNA HISLA increases resistance to apoptosis and aerobic glycolysis in breast cancer cells. Mechanistically, HISLA prevents PHD2 from interacting with HIF-1α and hinders HIF-1α’s hydroxylation and degradation. In turn, HISLA in macrophages is upregulated by lactate produced from glycolytic tumor cells, creating a feed-forward loop between tumor cells and tam. And restricting the release of HISLA from extracellular vesicles in breast cancer *in vivo* reduces glycolysis and chemoresistance (37). Meanwhile, Chen et al. demonstrated that the HIF-1α/glycolysis axis plays a role in FAM83A-AS1-mediated LA proliferation and metastasis, and the inhibition of tumor development and suppression of HIF-1α expression and glycolysis-related gene expression are seen *in vivo* with knockdown of FAM83A-AS1, making it a possible biomarker and treatment target for people with LA (38). However, given HIF-1α’s substantial influence on the expression of genes linked to the development of cancer and the inadequate effectiveness of chemotherapy, the future direction will be to create pharmaceuticals that target HIF-1 with more specificity by elucidating the molecular architecture of the structural domains that mediate HIF-1α’s critical activities.

#### c-MYC

Being a “master regulator” of cellular metabolism and cancer progression, c-MYC is among the most significant transcription factors in numerous types of cancer cells that are associated with reprogramming, proliferation, and resistance to chemotherapy. C-MYC exhibits both genetic and epigenetic changes in a wide variety of malignancies (39). There are reports indicating that lncRNAs influence the regulation of cancer energy metabolism via c-MYC post-translational modifications. Numerous lncRNAs exhibit substantial upregulation and serve as oncogenes within the colorectal or renal cancers, including the prostate cancer glycolysis-associated LncRNA SNHG7, the long intergenic noncoding RNA generated by energy stress FNCC1, mediated by the FoxO transcription factor, FILNC1, long intergenic noncoding RNA for IGF2BP2 stability (lncRNA LINRIS), and LINC00261. On a mechanical level, in prostate cancer PC-3 and DU-145 cells, SNHG7 stimulated glycolysis via the SRSF1/c-MYC axis; N6-methyladenosine (m6A) modification by methyltransferase-like 3 (METTL3) boosted SNHG7 stability (40).

Meanwhile, FILNC1 and LINRIS also regulate tumor cell proliferation. In renal tumors, knockdown of FILNC1 under circumstances of glucose deprivation only increases c-MYC protein levels and suggests that energy stress promotes the formation of tumors by regulating the FILNC1-auf1-c-MYC signaling axis through a minimum of two methods (41). In contrast, Wang et al. showed *in vivo* experiments that blocking LINRIS reduced the growth of tumors in both *in situ* and patient-derived xenograft (PDX) models. Mechanistically, LINRIS prevents IGF2BP2 from being stabilized by blocking its ubiquitination at the K139 site, a procedure that stops the autophagy-lysosomal pathway (alkaline phosphatase) from degrading IGF2BP2. Therefore, down-regulation of LINRIS mediated by MYC attenuates the downstream effects of IGF2BP2 and thus inhibits colorectal cancer (CRC) cell glycolysis (42). Furthermore, LINC00261 exerts its biological function by binding to miR-222-3p and triggering the HIPK2/ERK/c-MYC pathway. LINC00261 also inhibits the expression of c-MYC by sequestering IGF2BP1. It has been discovered that LINC00261 has unique epigenetic and post-transcriptional regulation mechanisms that act as tumor suppressors in pancreatic cancer, which could be beneficial for pancreatic cancer targeted treatment (43). In the moment, even though there are currently no c-MYC targeting medicines with any kind of clinical approval, more and more research is focused on addressing c-MYC targeting studies to develop viable approaches for treating tumors (44).

#### TGF-β1

Transforming growth factor-β1 (TGF-β1) exerts a wide range of activities during the process of developing an embryo, including differentiating cells and tissues, angiogenesis, wound healing, immunological response, and carcinogenesis. Several recent experiments have directly regulated tumor cell metabolism by mediating TGF-β1 through the glycolytic pathway. For example, TGF-β1 amplification stimulates the generation of ATP, lactate, and glucose absorption in primary hepatocellular carcinoma (HCC) cells. TGF-β1 serves as an upstream positive regulator of urothelial carcinoma-associated 1 (UCA1), which in turn is an upstream positive regulator of hexokinase-2 (HK2), and tumor-promoting lncRNA TGF-β1 tends to be up-regulated in tissues (45). Interestingly, quantitative reverse transcription polymerase chain reaction (qRT-PCR) results demonstrated that overexpression of exogenous lncRNA UCA1 upregulated HK2 and LDHA mRNA expression, whereas inhibition of lncRNA-UCA1 attenuated the glycolytic pathway, inhibiting prolactin (PRL) production and the proliferation of pituitary cancer cells (46). Meanwhile, the study has shown that LINC00973 has direct binding affinity towards LDHA, hence augmenting its enzymatic activity and facilitating glycolysis, ultimately leading to the proliferation of cancer cells (47, 48). Recent research indicates that LncDACH1 overexpression inhibits aberrant activation and collagen deposition induced by TGF-1, thereby preventing pulmonary fibrosis. Further, interestingly, the authors established that TGF-β1 facilitates the activation of cardiac fibroblasts, promotes the enhancement of glycolysis, and inhibits the expression of Linc00092. TGF-β1 is intimately associated with glycolysis and has the ability to modulate both lncRNA and related enzyme activity, making its regulation a very versatile and extensive study subject (49, 50). More information and mechanisms by which glycolysis-associated lncRNAs regulate cancer through transcription factors are shown in **Table 2**.

**Table 2:** Glycolysis-associated lncRNAs regulate cancer through transcription factors.

LncRNA	Level	Cancer type	Mechanisms	Glycolysis	Biological function	Ref.
CASC9	↑	Pancreatic cancer	AKT/HIF-1α	Activates	Proliferation, migration	(116)
FAM83A-AS1	↑	Lung adenocarcinoma	FAM83A-AS1/HIF-1α/glycolysis axis	Activates	Migration, invasion	(38)
RAET1K	↓	HCC	HIF1A/lncRNA RAET1K/miR-100-5p	Suppresses	Proliferation, invasion	(34)
SNHG7	↑	Prostate cancer	SRSF1/c-Myc	Activates	Proliferation	(40)
LINRIS	↑	Colorectal cancer	LINRIS-IGF2BP2-MYC	Activates	Proliferation	(42)
FILNC1	↓	Renal tumor	FILNC1–AUF1-c-Myc	Activates	Tumor growth, apoptosis	(41)
LINC00261	↓	Pancreatic cancer a	miR-222-3p/HIPK2/ERK/c-myc	Activates	Proliferation, migration, invasion	(43)
LINC00629 SLC2A1-DT	↓ ↑	Ovarian cancer HCC	HOXB4/LINC00629/c-Myc METTL3/SLC2A1-DT/β-catenin/c-Myc	Activates Activates	Proliferation, metastasis Proliferation, metastasis	(117) (118)
UCA1	↑	Hepatocellular Carcinoma	TGF-β1	Activates	Proliferation	(45)

The arrows denote upregulation or downregulation in cancer.

In summary, we found that the impact of glycolysis-associated transcription factors on tumors has received widespread attention. Transcription factors can regulate a variety of genes, even including some key enzymes, have important roles in metabolic shifts and the development of tumor cells, and can provide targets for the development of new tumor therapeutic strategies. Nevertheless, numerous challenges remain to be addressed when dealing with the intricate regulatory network. The interplay between various factors and the multifunctionality of transcription factors necessitate careful consideration. Additionally, the limitations imposed by detection technology pose a significant obstacle to the translation of these findings to clinical applications.

### Glycolysis-associated lncRNAs regulate cancer energy metabolism of metabolic enzymes

During cancer development, lncRNAs can also regulate many glycolysis-related enzymes (51). Key regulators of glycolytic enzymes by lncRNAs include 6-phosphofructokinase1, glucose-6-phosphate dehydrogenase, pyruvate kinase 2 (PKM2), LDHA, HK, and glucose transporter protein 1 (GLUT1). Thus, aberrant regulation and expression of glycolytic pathway enzymes are essential for tumorigenesis. For example, the lncRNA TMEM105, which is related to glycolysis, exhibits upregulation of LDHA and facilitates the liver metastasis of breast cancer by acting as a miR-1208 sponge (52). The manifestation of G6PD is enhanced by ELFN1-AS1 via the promotion of TP53 degradation, leading to the activation of the pentose phosphate pathway (PPP). Zhang et al. identified that cellular migration, invasion, and proliferation of esophageal cancer tumor cells were inhibited when ELFN1-AS1 was suppressed. This was achieved by inhibiting miR-183-3p, which upregulated glutamine-fructose-6-phosphate transaminase 1 (GFPT1) (53).

PKM2 is an essential metabolic enzyme that converts phosphoenolpyruvate to pyruvate, playing a vital role in cellular metabolism and tumor proliferation. In recent studies, in order to increase aerobic glycolysis, SNHG6 and hnRNPA1 are lncRNAs that interact to promote the growth of colorectal cancer by regulating alternative splicing of PKM. Meanwhile, LncRNA HULC modulates the enzymatic activities of LDHA and PKM2 by increasing their phosphorylation levels and enhancing their interaction with the intracellular structural domain of the upstream kinase FGFR1 (54, 55). Meanwhile, glycolysis-associated enzymes represented by PKM2 are diverse and mechanistically complex, and they are closely linked to transcription factors and profoundly influence material metabolism, functioning in interaction with multiple lncRNAs.

**Figure 2:**
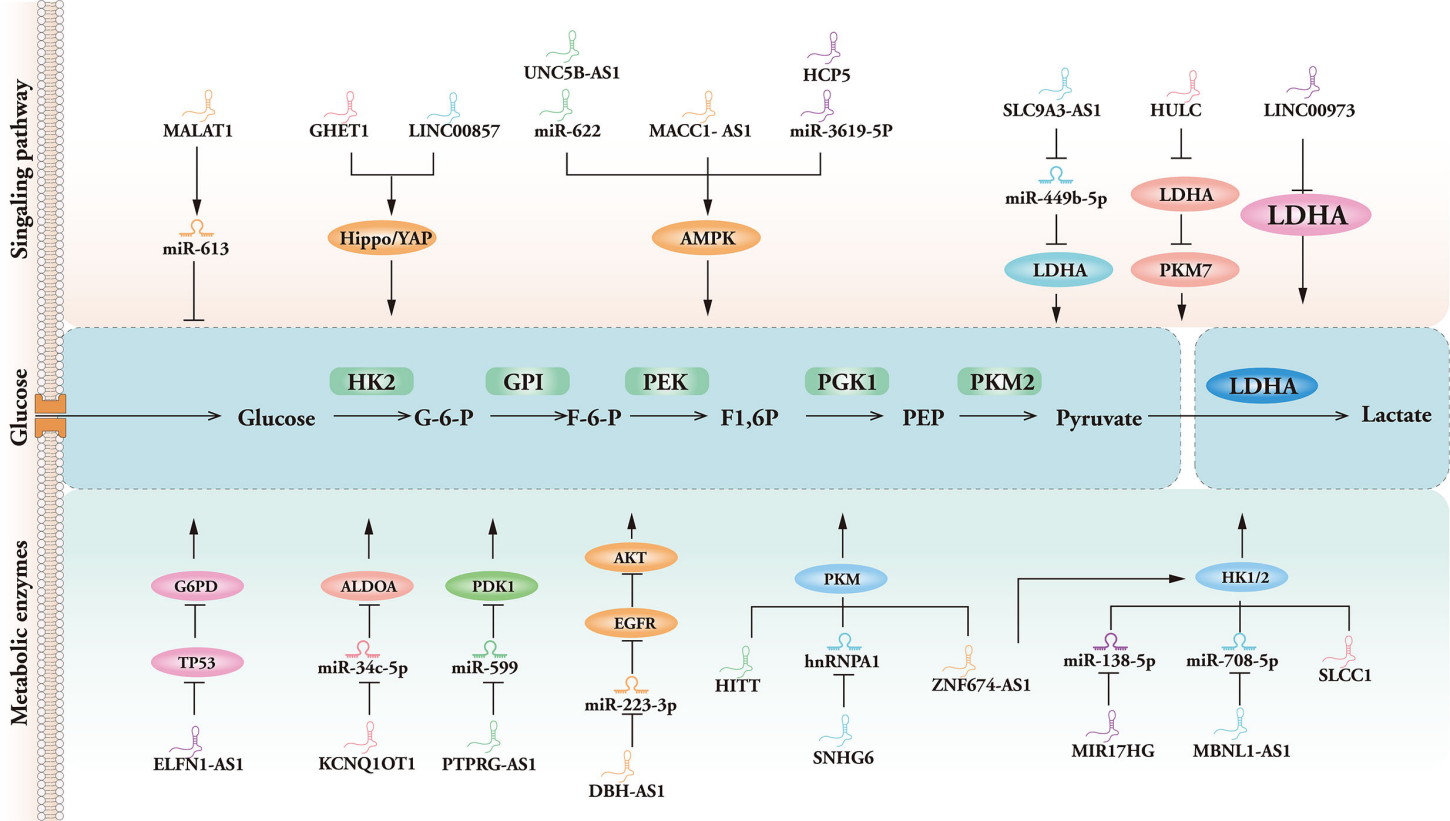
Mechanisms of glycolysis-associated LncRNA-mediated signaling pathways and enzymes of meditation in cancer.

Lactic acid, a major metabolite of glycolysis, has garnered considerable interest in recent years due to its role as a regulator that connects tumor progression and immunity. Zhao (56) et al. found that lncRNA HITT inhibited lactate production by suppressing the oligomerization of PKM2, thereby reducing tumor growth and macrophage polarization. Interestingly, hypoxic conditions have been found to drive the glycolytic activity of tumor-associated fibroblasts (CAF) through capillary dilatation ataxia mutated (ATM) oxidation, glucose transporter protein 1 phosphorylation, and PKM2 overexpression, and that lactic acid produced by CAF is ultimately used to drive breast cancer cell invasion through activation of the TGF1/p38 MAPK/MMP2/9 signaling axis and promotion of mitochondrial oxidative phosphorylation (57, 58). The Meanwhile, there exists a correlation between the spatial distribution of lactate concentration and the occurrence of lactation. Identifying several crucial lactation sites within the microenvironment of the tumor can aid in the recruitment of immune cells, induce modifications to the microenvironment, and collaborate with additional epigenetic modifications to stimulate tumorigenesis, progression, and direct modulation of gene expression associated with critical tumor pathways (59, 60). However, relevant studies are not focused enough and lack clinical validation, and further experimental data are urgently needed to prove their mechanisms. Additional research is warranted to explore the various functions and specific mechanisms underlying lactonization, as well as the regulatory enzymes involved in this process. Understanding the role of lactonization in exercise, lipolysis, neuroprotection, and angiogenesis is crucial, as it may offer important understandings for the creation of new medical diagnostics and treatment approaches for chronic diseases, including atherosclerosis and Alzheimer’s disease (61, 62). In a similar vein, it has been shown that lncRNAs such as UPF1, TUG1, and MALAT1 increase the activity of kinases connected to glycolysis, which has a facilitative effect on the glycolytic process (63). Meanwhile, from precancerous polyps to distant metastases, lncRNAs are frequently engaged in various phases of cancer and can be considered effective diagnostic biomarkers. More information and mechanisms by which glycolysis-associated lncRNAs regulate cancer through metabolic enzymes are shown in **Table 3**, **Figure 2**.

**Table 3:** Glycolysis-associated lncRNAs regulate cancer through metabolic enzymes.

LncRNA	Level	Cancer type	Mechanisms	Glycolysis	Biological function	Ref.
HULC	↑	Aerobic glycolysis	LDHA/PKM7	Activates	Proliferation	(55)
SNHG6	↑	Colorectal cancer	SNHG6/hnRNPA1/PKM↑	Activates	Proliferation, invasion and migration	(54)
CCAT1	↑	Gastric cancer	PTBP1/PKM2/glycolysis	Activates	Proliferation, migration, invasion	(119)
FEZF1-AS1	↑	Colorectal cancer	FEZF1-AS1/PKM2/ STAT3	Activates	Proliferation, metastasis	(120)
ZNF674-AS1	↓	Hepatic Carcinoma	HK2、PFKL、PKM2, GLUT1	Activates	Proliferation, invasion	(121)
495810	↑	Colorectal cancer	Ferulic Acid and P-Coumaric Acid/ lncRNA 495810/PKM2	Suppresses	Tumor growth	(122)
WFDC21P	↑	Hepatocellular carcinoma	Nur77-WFDC21P-PFKP/PKM2	Suppresses	Proliferation, tumor growth, metastasis	(123)
HITT	↓	Tumor	HITT-PKM2	Suppresses	Reduce tumor growth and macrophage polarization	(56)
LINC01852	↓	CRC	LINC01852/TRIM72/SRSF5/PKM2	Suppresses	Proliferation, chemoresistance	(124)
SNHG3	↑	castration-resistant prostate cancer	SNHG3 / miR-139-5p / PKM2	Activates	Proliferation, enzalutamide resistance	(125)
PWRN1	↓	HCC	PKM2	Suppresses	Proliferation	(126)
TMPO-AS1	↓	CRC	miR-1270/PKM2	Suppresses	Proliferation, migration, invasion	(127)
ELFN1-AS1	↑	Colorectal cancer	YY1 / ELFN1-AS1 / TP53 / G6PD	Activates	Proliferation, migration, invasion and suppresses apoptosis	(53)
SLC9A3-AS1	↑	Liver cancer	miR-449b-5p/LDHA	Activates	Proliferation, migration, invasion	(98)
LINC00973	↑	Cancer	LDHA/Warburg	Activates	Proliferation	(47)
KCNQ1OT1	↑	Osteosarcoma	kcnq10t1 /miR-34c-5p/ALDOA	Activates	Proliferation and suppressed apoptosis	(99)
SLCC1	↑	Colorectal cancer	SNP rs6695584/lncSLCC1/HK2	Activates	Tumor growth, progression.	(128)
RNCR2	↑	Melanoma	miR-495-3p/HK2	Activates	Proliferation, EMT	(129)
MIR17HG	↑	Colorectal cancer	miR-138-5p/HK1	Activates	Migration, invasion	(130)
MBNL1-AS1	↓	Liver cancer	miR-708-5p/ HK2/ HK1	Suppresses	Migration, invasion	(97)

The arrows denote upregulation or downregulation in cancer.

In conclusion, experimental investigations are increasingly focused on the post-transcriptional regulation of lncRNAs and the competitive binding of miRNAs to affect the activity and expression of enzymes, with a particular emphasis on the role of lactate in metabolism. While initial studies may have identified a correlation between a particular LncRNA and a glycolysis-related enzyme, elucidating its precise significance within the context of the entire biological process remains a formidable task. In the past couple of years, a growing multitude of investigations have furnished empirical evidence supporting the involvement of lncRNA in the pathogenesis of cancer (64). In a word, the interaction between lncRNAs and metabolic enzymes is extensive and complex, necessitating further investigation and scholarly inquiry to fully comprehend the specific mechanisms and interconnections involved.

## Multiple mediation of glycolysis-associated lncRNAs in tumor immune microenvironment

In the microenvironment of the tumor, the existence of immunological cells and immunological-related mediators plays an essential and pivotal role in facilitating tumor development (65, 66). Numerous research investigations have proven that glycolysis-associated lncRNAs are essential regulatory molecules that directly block the cancer immunity cycle, in addition to activating negative regulatory pathways to restrict tumor immunity. LncRNAs reshape the tumor microenvironment via the recruitment and activation of innate and adaptive lymphoid cells (67, 68). Subsequently, we concentrate on the correlation between glycolysis-associated lncRNAs, dendritic cells, T cells, B cells, and natural killer cells.

### Dendritic cells

The glycolytic pathway plays a crucial role in the activation and function of DC cells. Recent studies have found that DC cells enhance glycolytic activity when stimulated by signals in the cancer immune microenvironment. The function of lncRNAs in regulation produced from macrophages in the modulation of glycolysis in tumor cells has been shown, and host lncRNA MIR4435-2HG in primary myeloid dendritic cells (mDCs) from elite controllers (ECs) responded to TLR3-stimulated increases in oxidative phosphorylation and glycolytic activity by mTOR signaling pathway participants through the enhancement of immunometabolism activity of primary myeloid dendritic cells in EC by using specific epigenetic alterations on constituents of the mTOR signaling pathway (69). In contrast, it has been found that DC cells migration is inhibited by lnc-DPF3 produced by the CCR7 chemokine receptor through the inhibition of glycolysis mediated by HIF-1α. Furthermore, the critical role of lnc-DPF3 in impeding the late phase of dendritic cell migration and preventing inflammatory pathogenesis while preserving immune homeostasis is its delayed and induced function (70). As the understanding of the importance of lncRNAs and glycolysis in DC cell function increases, researchers are exploring the possibility of applying this knowledge to cancer immunotherapy. By targeting specific lncRNAs or modulating glycolytic pathways, it is possible to enhance the anti-tumor capacity of DC cells, thereby improving the outcome of cancer patients.

### T cells

Metabolic reprogramming is required for T cells to activate and perform their effector functions, and enhanced glycolysis can support T cells in high energy-demanding anti-tumor immune responses. Studies have shown that the cancer immunogenic lncRNA LIMIT, by targeting the LIMIT-GBP-HSF1 signaling axis, may restore MHC-I expression and function by cancer immunotherapy, which may also strengthen the T-cell-mediated immune response against cancers. As a result, lncRNA LIMIT may be considered an immunogenic target (71). Similarly, the researchers Zhao et al. made the observation that ovarian cancer cells exhibit elevated amounts of the microRNAs miR-101 and miR-26a. These microRNAs were shown to suppress the production of the methyltransferase EZH2. Consequently, EZH2 downregulation leads to glycolytic limitation in T cells and subsequent impairment of their functions (72). Meanwhile, numerous experiments have demonstrated that patients with grade II-III gliomas exhibit enhanced T lymphocyte infiltration, while Guo et al. used bioinformatics analysis and RT-qPCR to show that knockdown of lncRNA significantly reduces SHG-44 cell viability and proliferation and inhibits glycolysis, demonstrating the potential regulatory role of CRNDE in glucose metabolism in grade II-III gliomas. potential regulatory role of CRNDE in glucose metabolism in grade II-III gliomas (73–75). Furthermore, LncRNA NEAT1 plays a key role in the suppression of immune surveillance by T cells. NEAT1 is upregulated and positively modulates the expression of LDHA in prostate cancer. The anti-tumor effects are enhanced by the secretion of CD8+ T lymphocyte factors TNF-α, IFN-γ, and granzyme B, which is promoted by the knockdown of NEAT (76). The current study is still in its preliminary stages, but there is great potential for glycolysis-associated lncRNAs to be used in the cancer immune microenvironment. Future studies may further explore how these lncRNAs can be exploited clinically to enhance T cell-mediated anti-tumor immune responses, especially when used in combination with other immunotherapeutic approaches.

### B cells

Although there have been fewer studies on the direct regulation of B-cell glycolysis by lncRNAs, a number of studies have begun to reveal potential mechanisms in this area. For example, Xie et al. demonstrated that PDL1+ B cells were induced by the exosome lncRNA HOTAIR in order to inhibit antitumor immunity in colorectal cancer. Mechanistically, PDL1 expression on B cells was increased by exosome HOTAIR, which prevented ubiquitination degradation of the PKM2 protein and caused PKM2 to stimulate STAT3 transcription (77). Notably, extracellular miR-122 from hepatocytes can be delivered to hepatic stellate cells (HSCs) to regulate their proliferation and gene expression, and ectopic miR-122 expression inhibits BCL2 expression in human HSC (LX-2) cells (78). Meanwhile, Zhao et al. demonstrated that the MYC-regulated lncRNA NEAT1 promotes B-cell proliferation and lymphangiogenesis through the miR-34b-5p-GLI1 pathway in diffuse large B-cell lymphoma (79). These lncRNAs may indirectly affect the glycolytic process of B cells by regulating key enzymes of glycolysis or metabolic signaling pathways, thereby altering the behavior of B cells in the cancer immune microenvironment.

### Natural killer cells

**Figure 3:**
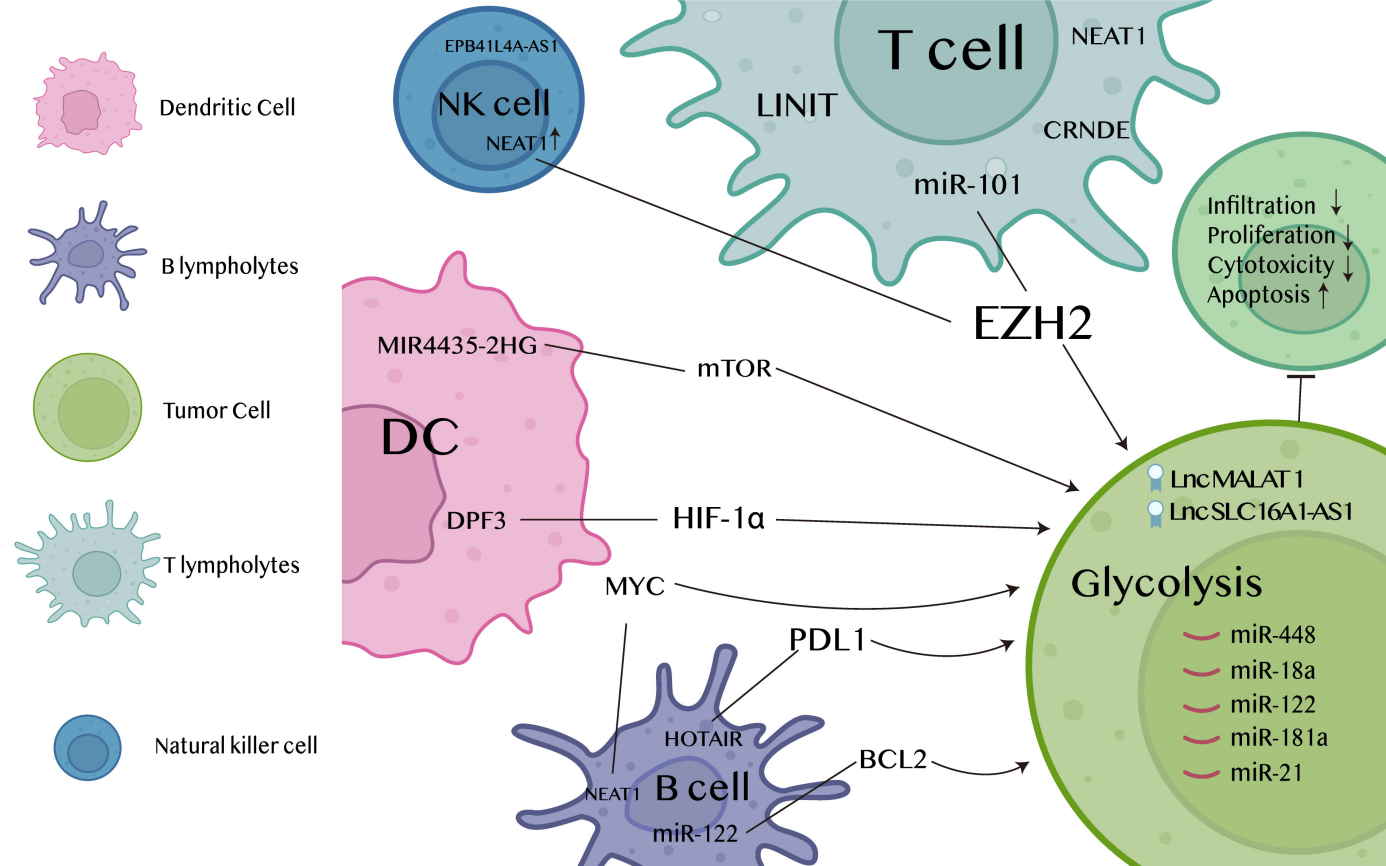
Mechanisms of glycolysis-associated lncRNAs regulating in tumor immune microenvironment.

The cytotoxic activity of NK cells can also be regulated by lncRNAs. NK cells are also one of the key immune components in the surveillance and eradication of neuroblastoma (NB). Within the neuroblastoma framework, data have demonstrated that the exosomal lncRNA EPB41L4A-AS1 translocates from CD56^bright^NK to CD56d^im^NK and inhibits glycolysis of target NKs. By means of exosomal transfer of the metabolism inhibitory lncRNA EPB41L4A-AS1, cross-talk between heterogeneous NK subpopulations was accomplished (80). Therefore, Exosomal lncRNA NEAT1 is up-regulated in multiple myeloma, and NEAT1 represses PBX1 by recruiting EZH2. Knockdown of PBX1 attenuates the effect of NEAT1-silenced exosomes on NK cells and multiple myeloma cells. Additionally, it enhances the expression of NKG2D, TNFα, and IFNγ in tumor tissues, hence promoting immune evasion by multiple myeloma cells (81). More information and mechanisms of glycolysis-associated lncRNAs regulating in tumor immune microenvironment are shown in **Figure 3**.

Notably, further research is warranted to determine whether lncRNAs regulate immune cell function synergistically, and future investigations may focus on identifying additional lncRNAs with potential combinatorial functions in immune cell behavior regulation.

## Risk models of glycolysis-associated lncRNAs in tumor immune microenvironment

An increasing number of lncRNA risk models for immunization have been developed in recent years to improve disease progression and provide clinical decision-making for cancer patients. Representative models, including those related to glycolysis, are a signature consisting of seven lncRNAs developed by Feng’s team, BC-associated LRLPS, and a multistep model of lncRNA-lncRNA cooperation that synergistically regulates functional modules. For example, a prognostic signature comprising seven immune-associated lncRNAs, namely AL391832.3, LINC00892, LINC02207, LINC02416, and PSMB8, demonstrated significant predictive capabilities for the survival outcomes of individuals diagnosed with ovarian cancer. These findings hold potential for offering valuable indicators for the implementation of clinical stratified management and personalized treatment selection for this specific patient population (82). Meanwhile, Li et al. constructed a lactate-associated lncRNA prognostic signature (LRLPS) for breast cancer patients and created a unique LRLPS that targets BC and has the ability to forecast treatment response, immunological state, and prognosis (83). By combining multi-omics data, Shao et al. developed a multi-step model of lncRNA-lncRNA collaboration based on synergistic regulation of functional modules and systematically constructed and analyzed a cooperative network of lncRNA-lncRNAs (IC-LncRNAs) spanning a wide range of tumors, which helped to clarify the similarities and distinctions among tumor mechanisms. The IC-LncRNAs synergistic regulation of T cell activation may bring novel approaches to immunotherapy for tumors (84, 85).

To summarize, glycolysis-associated LncRNAs have a significant impact on the immunological microenvironment of tumors and can be used to develop prognostic models based on their specificity. Recent results indicate that glycolysis is significantly engaged in tumor cells’ immunological evasion since it enhances immunosuppression and resistance to tumor drugs. This finding highlights the potential use of targeting glycolysis as a unique strategy in immunotherapy. The findings offer valuable theoretical evidence for further elucidating the connection between immunity and metabolism (86, 87).

## Implications of glycolysis-associated lncRNAs on tumor drug resistance

Chemotherapy is among the most critical therapeutic approaches in cancer treatment, and the glycolytic pathway of LncRNA transcription has been found to counteract tumor drug resistance (88, 89). For example, LncRNA GLTC promotes radioiodine resistance and the advancement of thyroid-like carcinoma via targeting increased succinylation and enzymatic activity of LDHA (90). The GLTC targets the enzymatic activity and succinylation of LDHA to facilitate the progression of thyroid cancer and promote resistance to radioiodine treatment.

According to recent studies, epigenetic regulation of different signaling molecules can reduce the sensitivity of cancer cells to cisplatin. LncRNA XIST, LncRNA-DANCR, and LncRNA-SARCC associated with LA promote chemoresistance through the glycolytic pathway. For example, it was demonstrated that suppressing the lncRNA XIST significantly decreased the expression of glycolysis-critical enzymes while increasing the expression of miR-101-3p, which reduced the rate of extracellular acidification, glucose uptake, and lactic acid products and increased lung cancer’s resistance to cisplatin. In contrast, the upregulation of the PKM2/PKM1 ratio via the LncRNA XIST/miR-137 axis was found to increase glycolysis and chemotherapy tolerance in colorectal cancer (91, 92). In the meantime, Shi et al. illustrated how LncRNA-DANCR disrupted the miR-125b-5p/HK2 axis, thereby inducing anaerobic glycolysis and desensitizing colorectal cancer cells to cisplatin (93). Interestingly, Wen et al. illustrated how LncRNA-SARCC could increase osteosarcoma sensitivity to cisplatin by targeting HK2 via mir-143 to inhibit glycolysis (94). Furthermore, there is an increasing body of data showing that elevated glycolysis rates are positively correlated with chemotherapy resistance, and the glycolysis of tumors has been identified as a prospective target for the creation of anticancer medications.

Several studies have shown evidence for the substantial participation of lncRNAs in the control of glucose metabolism via influencing the HIF signaling pathway, which can promote drug sensitivity. Chen et al. established that suppressing HISLA via interference-mediated targeting of Tam-specific lncRNA could potentially stabilize HIF-1α and offer a more practical method for inhibiting glycolysis and apoptosis resistance in tumor cells. This finding underscores the appeal of lncRNA as a target in the field of tumor therapy (37). Meanwhile, LncRNA HIF1a - AS1 facilitates the interaction between y-box binding protein 1 (YB1) and serine/threonine kinase (AKT) by regulating the AKT/YB1/HIF1a pathway, which promotes the phosphorylation of YB1 (pYB1) and enhances glycolysis, thereby contributing to gemcitabine resistance in pancreatic cancer (95, 96).

The resistance mechanism of cancer cells is not only related to chemotherapy but also inextricably linked to the targeted therapy of traditional Chinese medicine. Recent studies have shown that tretinoin lactone (TP) is implicated in the development of hepatocellular carcinoma. Nevertheless, the precise biochemical network that is controlled by TP remains unknown. LncRNAs MBNL1-AS1 and SLC9A3 play roles in various pathological progressions. In contrast to MBNL1-AS1, which is down-regulated in HCC tissues and increases the sensitivity of hepatocellular carcinoma to tretinoin via regulation of glycolysis mediated by miR-708-5p, the lncRNA SLC9A3-AS1 exhibits up-regulation in both hepatocellular carcinoma tissue samples and cells. Mechanistically, the inhibition of SLC9A3-AS1 resulted in a considerable reduction in the glycolysis rate mediated by the miR-449b-5p/LDHA axis, causing Huh7 cells’ susceptibility to TP to decline (97, 98).

Consequently, the function of glycolysis-associated lncRNA in the tumor immune microenvironment and the mechanisms of drug resistance in cancer cells are combined. The efficacy of targeting glycolysis has been shown by the regulation of tumor development and the augmentation of anti-cancer treatment. Finally, other non-coding RNAs associated with glycolysis, including lncRNAs and circRNAs, should additionally be investigated, as this would enable the enrichment of molecular networks that elucidate the etiology of malignancies (99). More information and mechanisms by which glycolysis-associated lncRNAs regulate cancer through other pathways are shown in **Table 4**.

**Table 4:** Glycolysis-associated lncRNAs regulate cancer through other pathways.

LncRNA	Level	Cancer type	Mechanisms	Glycolysis	Biological function	Ref.
SNHG5	↑	Breast cancer	miR-299/BACH1	Activates	Proliferation, invasion and migration	(131)
MIR31HG	↑	Colorectal cancer	MIR31HG-miR-361-3p -YY1	Activates	Proliferation, angiogenesis	(132)
SNHG5	↑	Breast cancer	SNHG5-miR-154-5p-PCNA	Activates	Proliferation, apoptosis	(133)
AP000695.2	↑	NSCLC	miR-335-3p/TEAD1	Activates	Proliferation, migration, EMT	(134)
UCA1	↑	Pituitary tumor	lncRNAUCA1-glycolysis-serum PRL axis	Activates	Inhibited growth and prolactin (PRL) secretion	(46)
NEAT1	↑	Prostate cancer-	NEAT1 / miR-98-5p / HMGA2	Activates	Proliferation	(76)
FGD5-AS1	↑	Breast cancer	hsa-miR-195-5p/NUAK2	Activates	Proliferation, migration, invasion	(135)
DLX6-AS1	↑	Neuroblastoma	miR-506-3p/STAT2	Activates	Proliferation	(136)
TUG1	↑	Liver cancer	miR - 524 - 5p/ SIX1	Activates	Migration, invasion	(137)
MCF2L-AS1	↑	Colorectal cancer	miR-874-3p/FOXM1	Activates	Proliferation, invasion	(138)
LncRNA-XIST/ microRNA-126	↑	Glioma	IRS1/PI3K/Akt	Activates	Migration, invasion, resistance to apoptosis	(139)
DDX11-AS1	↑	HCC	miR-195-5p/MACC1	Activates	Proliferation, migration, invasion, apoptosis	(140)
HOXB-AS3	↑	Epithelial ovarian cancer	miR-378a-3p/Wnt/β-catenin	Activates	Tumor growth, migration	(141)
Mir100hg	↑	Lung adenocarcinoma	Mir100hg/miR-15a-5p/31-5p/glycolytic	Activates	Metastasis	(142)
PTPRG-AS1	↑	Esophageal squamous cell carcinoma	miR-599 / PDK1	Activates	Proliferation, migration	(143)
UPF1	↓	Endometrial Cancer	PVT1/UPF1	Activates	Tumor growth, migration	(144)
DICER1-AS1	↓	Pancreatic cancer	YTHDF3/DICER1−AS1/DICER1/miR−5586−5p	Activates	Tumor growth, metastasis	(145)
EPB41L4A-AS1	↓	Cancer	HDAC2	Activates	Glutaminolysis	(146)
ZNF674-AS1	↑	Hepatocellular Carcinoma	miR-212-5p/FZD5/Wnt/β-	Suppresses	Proliferation, invasion	(121)
LET	↑	Esophageal Squamous Cell Carcinoma	miR-93-5p/miR-106b-5p/SOCS4	Suppresses	Proliferation, glutamine decomposition	(147)
HOTAIRM1	↓	NSCLC	miR-498/ABCE1	Suppresses	Proliferation, metastasis, apoptosis	(114)
H19	↓	Oral cancer	H19/miR-675-5p/PFKFB3	Suppresses	Proliferation	(105)
DBH-AS1	↓	Melanoma	miR-223-3p/EGFR/AKT	Suppresses	Proliferation, metastasis	(148)
SH3BP5-AS1	↑	HCC	miR-6838-5p/PTPN4	Activates	Proliferation, migration, invasion	(149)
NEAT1	↑	cervical cancer	WNT/β-catenin	Activates	Proliferation, migration, invasion	(13)
Linc01056	↓	HCC	PPARα	Suppresses	Sorafenib resistance	(150)
SNHG6	↑	Wilms' Tumor	miR-429/FRS2	Activates	Proliferation	(151)
LINC00665	↑	lung adenocarcinoma	let-7c-5p/HMMR	Activates	Proliferation	(12)

The arrows denote upregulation or downregulation in cancer.

## Other regulatory functions and clinical significance of glycolysis-associated lncRNAs

### Application of glycolysis-associated lncRNAs as biomarkers

Biomarkers contribute significantly to various fields such as personalized medicine, drug development, clinical care, and molecular breeding, which can expedite the process of medication development, serve as a preliminary marker of enhanced clinical responsiveness, and improve the level of patient safety. LncRNAs are an important direction of research at the molecular level (100, 101). Glycolysis-associated lncRNAs are more stable compared to proteins in cancer research and demonstrate a high degree of cell-tissue specificity and targeted therapeutic potential, constantly revealing new molecular regulatory networks. In contrast, in the treatment of cancer, protein molecules are susceptible to denaturation or inactivation by environmental conditions; some exogenous proteins or protein drugs may trigger an immune response, which can lead to adverse reactions or reduced efficacy; and protein molecules are structurally complex with limited targeting (102, 103).

Numerous lncRNAs are prospective biomarkers in the investigation of tumor cell suppression and glycolysis. LncRNA AFAP1-AS1 was also considerably expressed in patients with colon cancer. Patients exhibiting elevated levels of AFAP1-AS1 demonstrated a worse duration of survival and exhibited a strong association with tumor volume, local lymph node metastasis, disease stage, and overall survival. Accordingly, it is probable that LncRNA AFAP1-AS1 could function as a therapeutic target and diagnostic marker for colon cancer (104). In their study, Yang et al. observed a simultaneous upregulation of LncRNA H19 in both oral cancer cell lines and cancer associated fibroblasts (CAFs). The lncRNA H19/miR-675-5p/PFKFB3 axis promoted glycolysis in oral CAFs; lncRNA H19 inhibition in oral CAFs impeded glucose metabolism, cell proliferation, and migration. Furthermore, scientific investigations have revealed that lncRNA H19 influences tumor cell proliferation, metastasis, and apoptosis inhibition and that up-regulation of lncRNA H19 stimulates cancer cell resistance to chemotherapy and radiotherapy; thus, H19 not only provides new biomarkers for molecular diagnostics, but also serves as a new target for anti-tumor therapy (105–107). Furthermore, the lncRNA EGOT is frequently used to predict the prognosis of multiple or gastrointestinal tumors, and it potentially participates in numerous biological processes, such as autophagy and glycolysis (11, 108). Thus, despite the increasing convenience it provides, biomarker research remains far from complete and serves as a compass for future investigations into cancer treatments.

### Construction of prognostic models by glycolysis-associated lncRNAs

Since the diagnostic power of a single circulating lncRNA is not superior to that of a model consisting of multiple lncRNAs, an increasing number of prognostic models are undergoing growth with the advancement of bioinformatics, both for predicting disease progression as well as for early intervention and prevention, while providing new ideas and helping to make initial therapeutic decisions for cancer treatment (109, 110). Predictive models based on lncRNAs and clinical features are effective in predicting renal cell carcinoma with clear cells (111). Ma et al. constructed an alternative marker for clear cell renal cell carcinoma consisting of seven lncRNAs, which was able to precisely Predict the prognosis of individuals diagnosed with clear cell renal cell carcinoma, and there was a substantial correlation between distant metastasis, tumor size, stage, and grade. The experimental prognostic characteristics provide a foundation for tracking the effectiveness of mTOR inhibitors or learning more about how they work in renal cell carcinoma (112). In their study, Ma et al. created a unique bladder cancer prognosis model by examining nine lncRNAs associated with glycolysis. These lncRNAs are involved in various signaling pathways, including lymphocyte receptor signaling, the nuclear factor-k-gene binding pathway, the Notch signaling pathway, the P53 pathway, chemokine signaling, and oxidative phosphorylation, which provides a newer and more comprehensive approach for cancer research (112, 113).

### Therapeutic targeting of malignancy by glycolysis-associated lncRNAs

Inhibiting proto-oncogene expression decreases tumor cell growth and metastasis while increasing treatment susceptibility and apoptosis. In an *in vitro* investigation, scholars noted that lncRNA HOTAIRM1 silencing inhibited glucose consumption and lactate production in non-small cell lung cancer. Western blot analysis revealed that lncRNA HOTAIRM1 inactivation prevented the HK2 protein from being expressed, and the miR-498 inhibitor impeded this suppression. In another *in vitro* investigation, lncRNA KCNQ1OT1 was found to stimulate cell proliferation and inhibit apoptosis while promoting osteosarcoma cell development *in vivo*. lncRNA KCNQ1OT1 sponged miR-34c-5p in the capacity of a competitive endogenous RNA (ceRNA), hence inhibiting the development of osteosarcoma cells *in vivo*, and prevented the *in vivo* proliferation of osteosarcoma cells by directly targeting its 3’Untranslated. Region to inhibit aldolase A expression (99, 114). *In vivo* studies, knockdown of the lncRNAs MALAT-1, HULC, DANCR, and AP000695.2 revealed inhibition of tumor cell growth. Similarly, upregulation of oncogenes is an efficacious strategy to combat tumor development.

Moreover, the LncRNA ELFN1-AS1 has been observed to promote the proliferation of colorectal cancer cells and impede apoptosis by enhancing G6PD activity (53). In a separate investigation, Fusarium promotes carcinogenesis by enhancing glucose metabolism in colorectal cancer cells. One way Fusarium promotes lncRNA ENO1-IT1 transcription is by increasing the binding efficiency of transcription factor SP1 to the promoter region of this gene. Raise ENO1-IT levels alter histone modification patterns on ENO1 and other target genes by interacting with KAT7 histone acetyltransferase, thereby altering the biological function of colorectal cancer; therefore, targeting the pathway may be advantageous in the treatment of patients with CRC (11). Therefore, despite the increasing accessibility of biomarker research, it is still far from complete and serves as a compass for future cancer treatment research.

## Conclusions and discussions

Glycolysis has become more well-known in cancer research in recent years. LncRNAs play a significant role in the modulation of several crucial biological processes and tumor development through the glycolytic pathway, including regulating the manifestation of key enzymes of glycolysis, modulating lactate production and transport, engaging in glycolysis-associated immune escape from tumors, or forming complexes with transcription factors that regulate gene expression, which in turn affects the expression of related genes. Here we focus on the complex and multi-functional network of energy metabolism regulators controlled by post-translational modifications mediated by LncRNA. This network serves as a central node for numerous cell signaling pathways and is among the quickest ways in which cells react to both internal and external stimuli. Nevertheless, the present comprehension of its particular mode of action remains rather restricted, necessitating more investigation to clarify the complicated processes and regulatory pathways linked to its exact activity.

In conclusion, lncRNAs in tumors are similar to a regulatory key during glycolysis, and lncRNAs can turn on or off switches for key genes, signaling pathways, or cellular functions in cancer cells. They can be involved as regulators not only in the normal regulation of cells but also in immunity and drug resistance. The existence of such amazing regulatory keys has shed light on the mechanisms of cancer development, providing new ideas and potential therapeutic targets for early diagnosis, treatment, and prognostic evaluation of cancer. However, these molecular mechanisms and their interactions require further studies to elucidate.

### Perspectives

Regarding human genes, only 2% can code for proteins, of which 98% are non-coding sequences that form a complex regulatory network. A significant proportion of these DNA sequences, which lack protein-coding potential, undergo the process of transcription to become RNA molecules. This group includes a substantial number of microRNAs (miRNAs) as well as lncRNAs, numbering in the thousands for each category. As discussed herein, many studies have shown that lncRNAs act by regulating specific miRNAs downstream of them. However, individual lncRNAs are not limited to regulating only one miRNA, so it is critical to conduct a more extensive investigation into the myriad targets or signaling pathways that follow lncRNAs. As an illustration, LncRNA EGOT-mediated glycolysis also involves autophagy-associated regulation. Whether lncRNAs regulate these pathways necessitates additional research and discourse. On one side, the regulatory mechanisms behind these non-glycolytic reactions must be clarified; on the other side, clarifying the relationship between glycolysis and various non-glycolytic activities is of paramount importance so as to examine in greater detail how lncRNA regulation of tumor cell glycolysis affects the immune system’s monitoring mechanism for tumor cells. Further investigation of the intricate lncRNA-miRNA-mRNA competing endogenous RNA networks is warranted since they constitute a sophisticated and tightly controlled mechanism for governing gene expression and cellular processes and may help to address the etiology of diseases such as glioblastoma, osteoarthritis, neuroblastoma, heart disease, lung cancer, pancreatic cancer, and inflammation.

Early detection of cancer can increase survival or cure rates. Nevertheless, despite lncRNAs’ promising future as biomarkers in medical research, they still face challenges of uncertainty in functional interpretation, variability and dynamics in expression levels, standardization and technical differences, lack of validation and validation sets for clinical applications, and bioinformatics analysis and interpretation, and solving these problems requires additional research and methodological improvements. The use of multiple lncRNAs in model construction can greatly enhance diagnostic and predictive capabilities, but further extensive research is required in order to identify circulating biomarkers that possess the capability to consistently detect malignancies in their early stages.

## Glossary

AMPK: adenosine monophosphate-activated protein kinase
ATP: adenosine-triphosphate
CAF: cancer associated fibroblasts
CircRNAs: CircularRNA
c-MYC: cellular-myelocytomatosis viral oncogene
CRC: Colorectal cancer
DNA: Deoxyribonucleic acid
DC: Dendritic cells
ENO1: enolase 1
FILNC1: FoxO-induced long non-coding RNA 1
FoxO: Forkhead Box O
G6PD: Glucose-6-Phosphate Dehydrogenase
GLUT1: glucose transporter isoform 1
HCC: hepatocellular carcinoma
HIF-1α: hypoxia-inducible factor-1α
HISLA: HIF-1α-stabilizing lncRNA
HK2: hexokinase 2
HOTAIR: HOX antisense intergenic RNA
HOTAIRM1: HOX antisense intergenic RNA myeloid 1
HULC: highly up-regulated in liver cancer
IGF2BP2: insulin-like growth factor 2 mRNA-binding protein 2
LDHA: lactate dehydrogenase A
lincRNA: long intergenic noncoding RNA
LINRIS: long intergenic noncoding RNA for IGF2BP2 stability
Lnc RNA: Long non-coding RNA
lncRNA HITT: HIF-1α inhibitor at translation level
m6A: N6-methyladenosine
MACC1: metastasis-associated in colon cancer-1
MALAT1: metastasis-associated lung adenocarcinoma transcript 1
miRNA: microRNA
miRNA: MicroRNA: mRNA-binding, protein 2 stability (LINRIS) metastasis-associated in colon
mTOR: mammalian target of rapamycin
NADH: nicotinamide adenine dinucleotide
NADPH: nicotinamide adenine dinucleotide phosphate
NK: Natural killer cells
NSCLC: non-small cell lung cancer
OXPHOS: oxidative phosphorylation
PCa: prostate cancer
PFK1: phosphofructokinase-1;PFKFB3,6-phosphofructo-2-kinase/fructose-2,6-bisphosphatase3
PGK1: phosphoglycerate kinase 1
PHD2: proline hydroxylase domain 2
PI3K: phosphoinositide-3-kinase
PKM2: pyruvate kinase muscle isozyme M2
PKM2: Pyruvate kinase muscle isozyme M2
Tam: tumor-associated macrophages
TNBC: triple-negative breast cancer
TP53: Tumor Protein P53
UPF1: Up-frameshift protein 1
YAP: yes-associated protein
